# RETRACTED: He et al. The Multi-Station Fusion-Based Radiation Source Localization Method Based on Spectrum Energy. *Sensors* 2025, *25*, 1339

**DOI:** 10.3390/s25164976

**Published:** 2025-08-12

**Authors:** Guojin He, Yulong Hao, Yaocong Xie

**Affiliations:** 1 China Research Institute of Radiowave Propagation, Qingdao 266107, China; hegj@crirp.ac.cn; 2 School of Microelectronics, Tianjin University, Tianjin 300072, China; haoyulong98@tju.edu.cn; 3 School of Information and Control Engineering, Qingdao University of Technology, Qingdao 266200, China

The journal *Sensors* retracts the article titled “The Multi-Station Fusion-Based Radiation Source Localization Method Based on Spectrum Energy” [1], cited above.

Following publication, concerns were brought to the attention of the Editorial Office regarding the validity of a number of citations present in this publication [1].

Adhering to our standard procedure, an investigation was conducted by the Editorial Office, and the Editorial Board was unable to verify the sources of a significant number of references cited in this publication. Following discussions, the authors outlined further concerns relating to a data retrieval error that impacted the accuracy of the data presented in Table 3. As a result, the Editorial Board has lost confidence in the reliability of the findings and has decided to retract this publication [1], in accordance with MDPI’s retraction policy.

This retraction was approved by the Editor-in-Chief of the *Sensors* journal.

The authors agreed to this retraction.

## References

[1] He G., Hao Y., Xie Y. (2025). RETRACTED: The Multi-Station Fusion-Based Radiation Source Localization Method Based on Spectrum Energy. Sensors.

